# Brief report: a comparison of child mental health inequalities in three UK population cohorts

**DOI:** 10.1007/s00787-019-01305-9

**Published:** 2019-03-08

**Authors:** Stephan Collishaw, Emma Furzer, Ajay K. Thapar, Ruth Sellers

**Affiliations:** 1 Child and Adolescent Psychiatry, Division of Psychological Medicine and Clinical Neurosciences, MRC Centre for Neuropsychiatric Genetics and Genomics, Cardiff University School of Medicine, Hadyn Ellis Building, Maindy Road, Cardiff, CF244HQ Wales UK; 2 Rudd Centre for Adoption Research and Practice, School of Psychology, University of Sussex, Brighton, England UK

**Keywords:** Inequality, Child mental health, Time trends, Cohort

## Abstract

There are substantial health disparities between children from low and higher income families. The study aimed to test changes in child mental health inequalities across three large UK population cohorts of 11-year-old children assessed in 1999, 2004 and 2012 as part of the British Child and Adolescent Mental Health Surveys and Millennium Cohort Study. Child mental health was assessed using parent and teacher versions of the Strengths and Difficulties Questionnaire. There were substantial differences in parent and teacher reported symptom scores between children from low and higher income families in each cohort. Differences in parent-reported symptoms increased over time (ES 0.35 [95% CI 0.20, 0.49] in 1999, ES 0.39 [95% CI 0.17, 0.61] in 2004, ES 0.54 [95% CI 0.49, 0.58] in 2012); cohort interaction: *p* = 0.01). This study found that marked child mental health inequalities exist. The mental health gap between advantaged and disadvantaged children has not reduced over the last 20 years and may be getting worse.

## Introduction

Alleviating inequalities in child health is a major policy priority internationally, including for successive UK governments [1, 2]. However, evidence highlights stark and growing inequalities in child physical health [3]. Less is known about inequalities affecting children’s mental health and whether these have changed over time, but there is some evidence that inequalities in adolescent emotional problems increased in the second half of the twentieth century [4]. Our aim here was to test family income inequalities in child mental health problems in the UK in the twenty-first century.

## Methods

Three large unselected UK population cohorts of 11-year-old children were compared: 1999 British Child and Adolescent Mental Health Survey (BCAMHS 1999, *N* = 928), 2004 British Child and Adolescent Mental Health Survey (BCAMHS 2004, *N* = 670) and the Millennium Cohort Study (MCS) 2012 sweep (*N* = 11,474). The use of identical symptom screens allows for valid ‘like-with-like’ comparison of the prevalence of child mental health problems across unselected population cohorts [5], and parents and teachers provide unique and complementary information about children’s mental health [6]. Parents and teachers in each study completed the widely used and extensively validated Strengths and Difficulties Questionnaire (SDQ) to assess mental health [7]. The SDQ total score includes 20 items assessing conduct problems, emotional problems, hyperactivity, and peer problems. For cross-cohort comparisons of mental health inequalities, parent- and teacher-rated total SDQ scores were each standardised within each cohort (mean = 0, SD = 1) to allow direct comparison of effect sizes (i.e. SD unit differences). Parents provided information about gross household income, and analyses compared families below or above the bottom quintile of incomes within each cohort. Standard survey-specific weights accounted for non-response and sample stratification (MCS). Linear regression models tested differences in SDQ total scores within each cohort, and cross-cohort comparisons (using standardised SDQ outcome scores) tested the interaction between cohort and low family income.

## Results

**Fig. 1:**
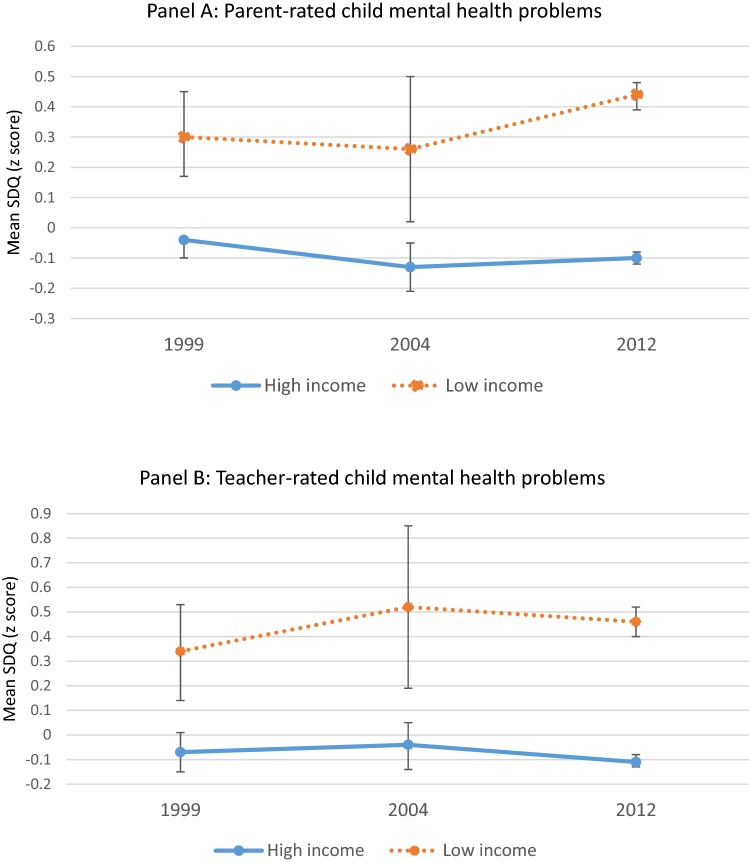
Standardized mental health problem scores for three nationally representative samples of 11-year-old children from high and low-income families. Mean *z* scores and 95% CIs. (**a** parent reports; **b** teacher reports)

There was no overall change in levels of child mental health problems reported by parents (total SDQ symptom scores: 1999 = 8.34 [95% CI 7.98, 8.70], 2004 = 7.85 [7.40, 8.31], 2012 = 8.12 [8.02, 8.23], *p* = 0.29) or by teachers (1999 = 6.15 [95% CI 5.72, 6.57], 2004 = 6.13 [5.59, 6.66], 2012 = 5.92 [5.80, 6.05], *p* = 0.48). There were substantial income inequalities in child mental health within each cohort (see Fig. 1). In addition, there was a significant cohort by income interaction for parent reports (beta = 0.074 [95% CI 0.020, 0.176], *p* = 0.01) showing that income inequalities varied across the study period. Comparing mental health problem scores for children from low and higher income families in each cohort, we observed that effect sizes increased by 54% across the study period (ES 0.35 [95% CI 0.20, 0.49] in 1999, ES 0.39 [95% CI 0.17, 0.61] in 2004, ES 0.54 [95% CI 0.49, 0.58] in 2012). For teacher reports, there were substantial differences between poor and non-poor children within each cohort (ES 0.41 [95% CI 0.23, 0.59] in 1999, ES 0.56 [95% CI 0.22, 0.90] in 2004, ES 0.57 [95% CI 0.51, 0.62] in 2011). The cohort by income interaction tended in the same direction, but was not significant (beta = 0.055 [95% CI − 0.013, 0.165], *p* = 0.09).

## Discussion

Successive UK governments have committed to reducing inequalities in child health and to improving children’s mental health [1, 2]. However, inequalities in children’s physical health are growing [3], and our study confirms that there are also substantial inequalities in child mental health. The mental health gap between advantaged and disadvantaged children is not reducing and may be getting worse. This has important implications for the future given that more than half of all adult mental disorders are preceded by mental health problems in childhood [8]. These findings on their own do not address reasons for inequalities in mental health, but evidence from intervention and quasi-experimental designs suggests that family poverty does likely have causal effects on child mental health [9, 10]. There have been substantial public spending cuts in many European countries, including the UK, which over the past decade have disproportionately impacted household incomes, financial security, public support, health programs and mental health services for the most disadvantaged families in society [11]. The findings represent an urgent call to action to close the substantial gap in mental health between poor and better off children, to prioritise social policy that mitigates the impacts of public spending cuts on the most vulnerable families in society, and to make appropriate adjustments in service provision in community and health care settings to support the mental health of children from low-income families.
